# A dual-platform toolkit for transient gene expression and genome editing in rubber dandelion (*Taraxacum kok-saghyz*)

**DOI:** 10.1016/j.abiote.2026.100053

**Published:** 2026-05-13

**Authors:** Xinbo Li, Rundong Shen, Xuesong Cao, Mugui Wang, Jian-Kang Zhu, Yifu Tian

**Affiliations:** a Ministry of Agriculture and Rural Affairs Key Laboratory of Gene Editing Technologies (Hainan), Institute of Crop Sciences and National Nanfan Research Institute, Chinese Academy of Agricultural Sciences, Sanya, 572024, China; b Yazhouwan National Laboratory, Sanya, 572024, China; c Institute of Advanced Biotechnology, and School of Medicine, Southern University of Science and Technology, Shenzhen, 518055, China

**Keywords:** *Taraxacum kok-Saghyz*, Protoplasts, Transient expression, Subcellular localization, Genome editing, CRISPR/Cas9, Base editing

## Abstract

Transient gene expression in mesophyll protoplasts is a valuable approach for investigating gene function, plant physiological processes, and molecular mechanisms. Rubber dandelion (*Taraxacum kok-saghyz*, TKS) is an ideal model for studying rubber biosynthesis and serves as a promising source of natural rubber and inulin. However, developing efficient protoplast-based systems for TKS remains challenging. In this study, we established a robust method for isolating mesophyll protoplasts from TKS by optimizing enzymatic conditions for cell wall digestion. We subjected the protoplasts to PEG/calcium-mediated transfection and evaluated promoter activities, expressed and detected target proteins, confirmed the subcellular localization of the target proteins, and examined transcription factor–DNA interactions under physiological conditions. We also developed a rapid assessment strategy for genome-editing tools in TKS protoplasts using multiple reporter systems. We evaluated these optimized tools in a tissue-culture-free hairy root transformation system, establishing a dual-platform toolkit for functional genomics in TKS. This work provides an efficient approach for TKS protoplast preparation, facilitating studies of gene function and advancing biotechnological research in this rubber-producing crop.

## 1 Introduction

Rubber dandelion (*Taraxacum kok-saghyz*, TKS) is a perennial herbaceous plant native to China and Kazakhstan [1]. TKS roots contain up to 20% (dry weight) natural rubber composed of *cis*-polyisoprene, resembling that of the Brazilian rubber tree. Additionally, TKS roots are rich in inulin (25–40% dry weight), the primary storage form of carbohydrates, with significant applications in the food and non-food industries and for biofuel production [2]. The high rubber content, ease of cultivation, environmental adaptability, and short maturation period of TKS make it a promising alternative source of natural rubber, with substantial strategic importance. Although a simplified genetic transformation system for TKS has been established [3,4], efficient transient expression systems are still needed for the rapid validation of gene function and for the optimization of biotechnological tools for this plant.

Protoplasts are cells whose cell walls have been enzymatically removed, creating a single-cell system ideal for studying signaling pathways, metabolism, and gene expression. Protoplasts are excellent systems for investigating gene function and molecular mechanisms [5] and are widely used to study the post-translational modifications and subcellular localizations of proteins. For instance, transient gene expression in *Arabidopsis* (*Arabidopsis thaliana*) protoplasts revealed that RAH1 regulates resistance to aluminum toxicity and growth by mediating the ubiquitination and degradation of the transcription factor STOP1 [6]. Single-molecule localization imaging of protoplasts demonstrated that activated ZAR1 forms a pentameric resistosome at the plasma membrane, shedding light on its role in triggering immune responses and cell death [7]. Protoplasts are also valuable for identifying protein interactions. Screening avirulence (*Avr*) gene libraries through transient expression of known resistance (*R*) genes in wheat (*Triticum aestivum*) protoplasts facilitated the identification of interacting *R-Avr* pairs [8]. Additionally, analysis using carrot (*Daucus carota* ssp. sativus) protoplasts provided key insights into the mechanisms underlying host-hopping by RNA viruses across fungi, plants, and animals [9].

The CRISPR-based genome-editing toolkit has revolutionized both fundamental and applied biological research over the past decade [10]. Efficient protocols for rapidly evaluating and optimizing genome editors in protoplasts have been developed for multiple plant species. Prime editors have been optimized by modifying codons, selecting different promoters, and adjusting editing conditions in rice (*Oryza sativa*) and wheat, enabling the creation of precise point mutations, insertions, and deletions [11]. Protoplast-based genome editing facilitated seamless gene fragment replacement in tobacco (*Nicotiana tabacum*) [12] and the creation of transgene-free *Salvia miltiorrhiza* with enhanced bioactive compounds [13]. Furthermore, CRISPR interference (CRISPRi)-based synthetic gene circuits have been constructed in *Physcomitrium patens*, wheat, and *Brassica napus* protoplasts, establishing a modular system with multiple logic gates to regulate gene expression [14].

Although highly successful protoplast isolation and transient transformation protocols have been established for common dandelion (*Taraxacum officinale*) [15], a species closely related to TKS, these methods have not yet been optimized for high-throughput or downstream genomic studies. Moreover, the abundant laticifers and secondary-metabolite profile of TKS [2] introduce additional layers of complexity that require the further refinement of these protocols for this plant. Consequently, a robust, reproducible protoplast system tailored to TKS remains to be established.

Building on this foundational work, in the current study, we established an efficient, high-yielding method for viable protoplast isolation from TKS leaves by systematically optimizing enzymatic conditions. We applied this system to a range of downstream analyses, including promoter screening, protein expression, subcellular localization analysis, and transcriptional regulation assays. Furthermore, we employed TKS protoplasts for functional validation and to compare the efficiencies of multiple CRISPR/Cas9-based editors, establishing this system as a powerful platform for analyzing gene function and optimizing editing tools for this crop. Finally, we further validated the editing tools optimized in the protoplast system in a tissue-culture-free hairy root transformation system, providing a complementary platform for stable, root-specific validation of gene function in this rubber-producing crop.

## 2 Results

### 2.1 Optimization of enzymatic conditions for TKS protoplast isolation

**Fig. 1:**
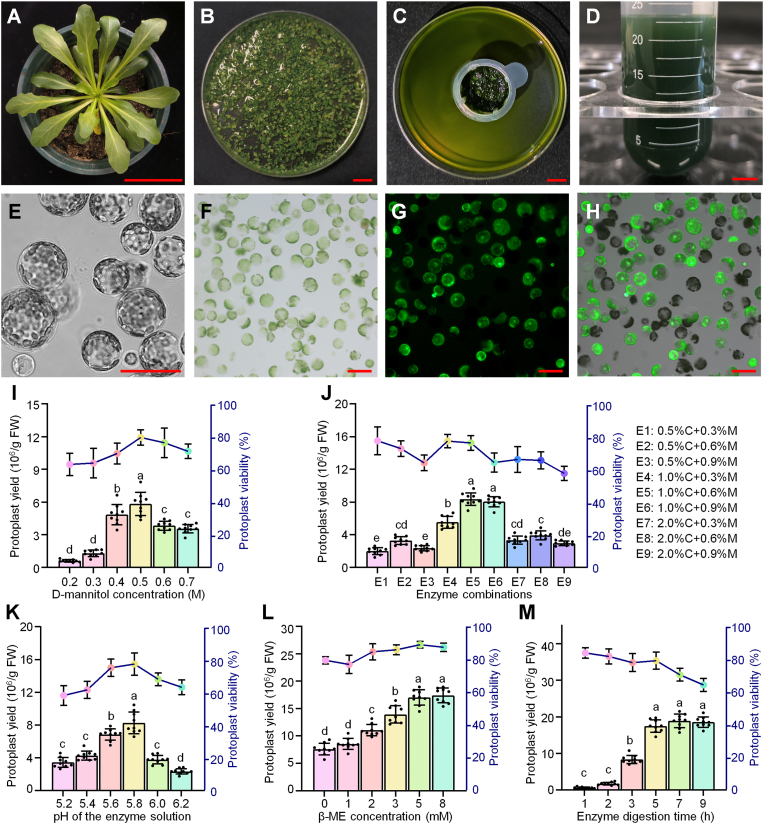
Optimization of the enzymatic conditions for TKS protoplast isolation. **A-G** Protoplast preparation and assessment: (A) *Taraxacum kok-saghyz* seedling, bar = 5 cm. (B) Leaves cut into small pieces in enzyme solution, bar = 1 cm. (C) Removal of plant tissue using a 70 μm filter after enzymatic digestion, bar = 1 cm. (D) Collection of protoplasts by centrifugation in a 50 mL round-bottomed tube, bar = 1 cm. (E) Protoplast morphology visualized by differential interference contrast (DIC) microscopy, bar = 50 μm. (F) Bright field images of protoplasts captured under a microscope, bar = 50 μm. (G) Assessment of protoplast viability by FDA staining, bar = 50 μm. **H** Overlay of bright field (F) and FDA fluorescence (G) images, bar = 50 μm. **I–M** Optimization of protoplast isolation conditions: (I) D-mannitol concentration, (J) enzyme composition, (K) pH of the enzyme digestion solution, (L) addition of β-mercaptoethanol, (M) enzyme digestion time. Protoplast yield (bar graphs) was determined by cell counting, and viability (line graphs) was assessed by FDA staining. For (I–M), data are presented as the mean ± standard deviation (SD) of nine replicates. Different letters (a-e) denote statistically significant differences (*P* < 0.05), as determined by one-way ANOVA.

To obtain TKS protoplasts with good physiological properties, we collected leaves from 4-week-old transplanted seedlings and finely chopped them using a double-edged razor blade (Fig. 1A and B). Following enzymatic digestion, protoplasts were released into the enzyme solution, turning the solution green as the leaf tissue degraded. We collected the protoplasts by filtration, transferred them to centrifuge tubes (Fig. 1C and D), and examined them under a microscope to assess their morphology and integrity (Fig. 1E). To measure protoplast viability, we calculated the ratio of FDA-stained cells to total cells (Fig. 1F–H).

We conducted a detailed investigation to evaluate factors influencing the preparation of TKS protoplasts. To balance osmotic pressure and minimize protoplast rupture during enzymatic digestion, we tested a gradient of D-mannitol concentrations ranging from 0.2 M to 0.7 M in the enzyme solution. The osmotic pressure of the enzyme solution significantly affected protoplast yield, with the highest yield (5.8 × 10^6^/g FW) obtained using 0.5 M D-mannitol (Fig. 1I). Subsequent optimizations were performed using this concentration.

To determine the optimal concentrations of cellulase and macerozyme for TKS protoplast isolation, we tested a gradient combination of these enzymes. Gradually increasing the enzyme concentration resulted in higher protoplast yields, with the highest yield (8.4 × 10^6^/g FW) achieved at 1.0% (w/v) cellulase R10 and 0.6% (w/v) macerozyme R10 (Fig. 1J). When the enzyme concentrations exceeded this optimal level, the protoplast yield decreased.

The optimal pH range for enzymatic digestion was between 5.6 and 5.8 (Fig. 1K). Adding 5 μM of the reducing agent β-mercaptoethanol (β-ME) to the enzyme solution maintained a high protoplast yield (1.7 × 10^7^/g FW) (Fig. 1L). Additionally, a digestion time of 5 h was determined to be optimal, balancing efficient preparation with proper TKS protoplast activity (Fig. 1M).

Therefore, the optimal enzyme solution used to prepare TKS leaf protoplasts is as follows: 0.4 to 0.5 M D-mannitol, a mixture of 1.0% cellulase R10 and 0.6% macerozyme R10, pH 5.6 to 5.8, and 5 mM β-ME. Combined with a 5-h digestion time, this optimized protocol yields sufficient amounts of healthy TKS protoplasts, providing an effective platform for studying intracellular processes and elucidating signaling pathways.

### 2.2 Efficient gene expression in TKS protoplasts using the 2 × 35S promoter

**Fig. 2:**
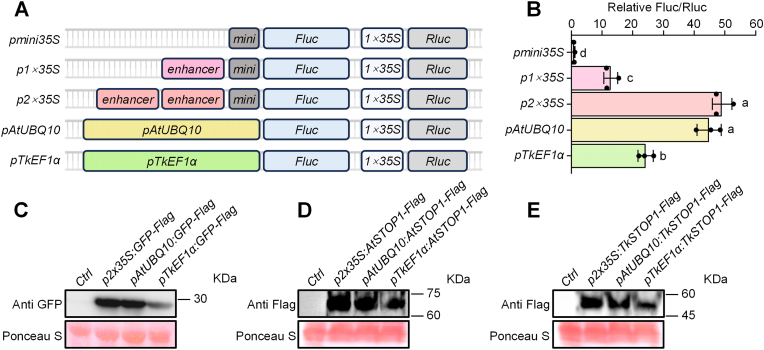
Efficient gene expression in TKS protoplasts using the *2 × 35S* promoter. **A** Diagram of the dual-luciferase reporter system. *Rluc* driven by the standard *35S* promoter served as an internal reference, while the test promoters were used to drive *Fluc* expression. **B** Relative luminescence intensity obtained by calculating the ratio of Fluc to Rluc signals. Data are presented as the mean ± standard deviation (SD) of three independent biological replicates. Each data point represents one replicate, and different letters (a-d) indicate statistically significant differences (*P* < 0.05), as determined by one-way ANOVA. **C** Detection of GFP accumulation in TKS protoplasts by immunoblotting. Target proteins were detected using GFP antibody, with total protein loading indicated by Ponceau S staining. **D–E** Detection of AtSTOP1-Flag (D) and TkSTOP1-Flag (E) accumulation in TKS protoplasts by immunoblotting. Target proteins were detected with a Flag antibody, and equal loading was confirmed by Ponceau S staining.

To optimize the efficiency of transient gene expression in TKS protoplasts, we evaluated several promoters using a dual-luciferase assay. The *Renilla* luciferase gene (*Rluc*), driven by the *cauliflower mosaic virus* (*CaMV*) *35S* promoter, served as an internal reference, while firefly luciferase (*Fluc*) expression was driven by various promoters (Fig. 2A). After cloning the promoters into the pDual-LUC vector and transiently expressing them in TKS protoplasts, we compared their promoter activities based on relative luminescence intensity. We assessed the activities of different versions of the *35S* promoter (Fig. S1A) [16,17], the *Arabidopsis AtUBQ10* promoter (Fig. S1B), and the TKS endogenous promoter *TkEF1α* (Fig. S1C) in TKS protoplasts. The *mini35S* promoter, a truncated 46 bp version of the *35S* promoter, drove low transcriptional activity in TKS protoplasts. By contrast, the *1* × *35S* promoter, which is the standard *35S* promoter composed of *mini35S* and an enhancer, exhibited 12.8-fold higher activity than the *mini35S* promoter. The *2* × *35S* promoter, which contains two tandemly repeated enhancers flanking the *mini35S* core, showed the highest activity, reaching levels 49.1-fold higher than those of the *mini35S* promoter and comparable to those of the *AtUBQ10* promoter. The endogenous promoter *TkEF1α* displayed 1.9-fold higher activity than the *1* × *35S* promoter but only approximately half the activity of the *2 × 35S* promoter (Fig. 2B).

Green fluorescent protein (GFP; derived from the jellyfish *Aequorea victoria*) and its variants are widely used to label target proteins, allowing temporal and spatial changes in these proteins to be observed and tracked [18]. We therefore employed GFP to evaluate the activities of the candidate promoters. In an immunoblot assay, GFP signals were significantly stronger when driven by the *2 × 35S* or *AtUBQ10* promoters compared to the *TkEF1α* promoter (Fig. 2C), indicating that the *2 × 35S* and *AtUBQ10* promoters are more effective in driving strong gene expression in TKS protoplasts. We also attempted to express homologs of *STOP1*, encoding a key transcription factor involved in resistance to acid soil syndrome, in TKS protoplasts [19]. Based on the published genome and gene annotation of TKS [1], we identified four STOP1-like genes. Phylogenetic analysis revealed that *TkSTOP1* is the ortholog of *Arabidopsis AtSTOP1*, whereas *TkSTOP2a*, *TkSTOP2b*, and *TkSTOP2c* are more closely related to *Arabidopsis AtSTOP2* (Fig. S2A). We used SMART software to predict the functional domains of the encoded proteins. All STOP1-like proteins are predicted to contain four C2H2 zinc finger domains. However, unlike STOP1, STOP2 and its homologs lack extended C-terminal regions (Fig. S2B). We expressed *AtSTOP1* and *TkSTOP1* in the pSAT6 backbone in our TKS protoplast transient expression system (Fig. S2C). Expression driven by the *TkEF1α* promoter was significantly lower than that driven by the *2* × *35S* promoter (Fig. 2D), which is consistent with the lower expression levels observed for exogenous proteins such as GFP (Fig. 2C). These results demonstrate that the *2* × *35S* promoter is highly effective in driving stable target gene expression in TKS protoplasts, highlighting its potential as a powerful tool for gene expression studies and genetic engineering in TKS.

### 2.3 Efficient protein localization in TKS protoplasts

**Fig. 3:**
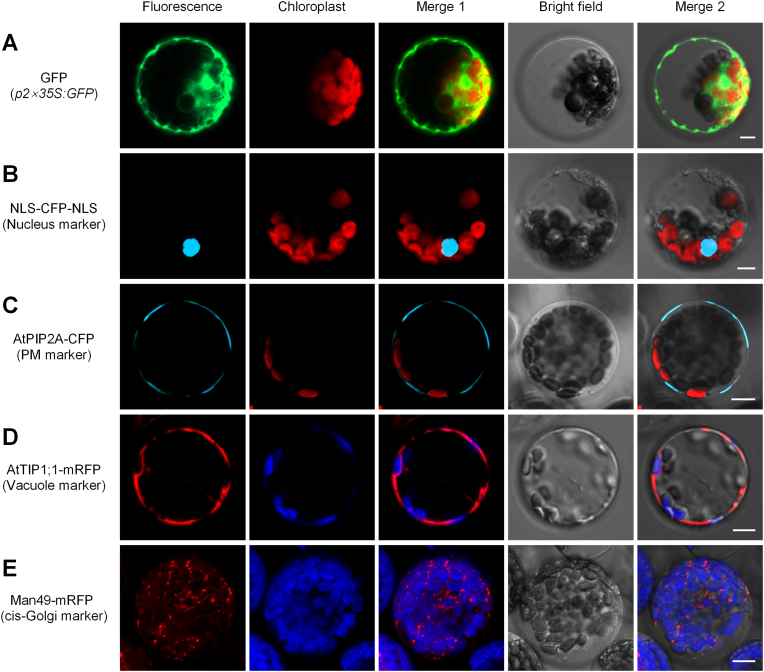
Efficient protein localization in TKS protoplasts. Images illustrate the transient expression of various fluorescent protein markers in TKS protoplasts, demonstrating the feasibility of using TKS protoplasts for the subcellular localization of proteins. **A** GFP expression in protoplasts as a control for general expression. **B** NLS-CFP-NLS, a nucleus localization marker. **C** AtPIP2A-CFP, a plasma membrane localization marker. **D** AtTIP1; 1-mRFP, a vacuolar membrane localization marker. **E** Man49-mRFP, a *cis*-Golgi localization marker. Bars = 10 μm.

Transient expression in protoplasts is a valuable approach for validating the intracellular localizations of proteins. Using our optimized transient expression system in TKS protoplasts, we developed an efficient method for assessing the subcellular localizations of exogenous proteins. *GFP* expression under the control of the *2* × *35S* promoter in TKS protoplasts resulted in a transformation efficiency of 56.3% (Fig. S3). The GFP fluorescence signals in TKS protoplasts showed both cytoplasmic and nuclear localizations, a pattern consistent with that observed in protoplasts from other plants (Fig. 3A).

We constructed a nuclear localization marker by fusing two different nuclear localization signals (NLS) to the N-terminus and C-terminus of CFP (cyan fluorescent protein), a variant of GFP, using the pSAT6 backbone. The N-terminal SV40 NLS (APKKKRKVGIHGVPAA) was derived from simian virus 40 (SV40), which features the characteristic lysine- and arginine-rich core motif “PKKKRKV”, while the C-terminal bipartite nucleoplasmin NLS “KRPAATKKAGQAKKKK” was derived from nucleoplasmin to strengthen nuclear import of CFP (Fig. S4). When fused with these NLS sequences, CFP exhibited typical nuclear localization in TKS protoplasts (Fig. 3B).

We constructed a plasma membrane localization marker by fusing AtPIP2A, a plasma membrane-localized PIP (plasma membrane intrinsic protein) aquaporin from *Arabidopsis* [20], to the N-terminus of CFP using the pSAT6 backbone (Fig. S5A and C). The AtPIP2A-CFP fusion protein was successfully expressed in TKS protoplasts and localized to the plasma membrane, clearly enveloping chloroplasts (Fig. 3C). In addition, we generated a vacuolar membrane localization marker by fusing the γ-TIP (tonoplast intrinsic protein) aquaporin AtTIP1;1 from *Arabidopsis* [21] to the N-terminus of mRFP (monomeric red fluorescent protein) (Fig. S5B and D). The AtTIP1; 1-mRFP fusion protein was successfully expressed in TKS protoplasts and localized to the vacuolar membrane, with chloroplasts positioned at its periphery (Fig. 3D). We also constructed a *cis*-Golgi localization marker by fusing a segment of soybean (*Glycine max*) α-1,2-mannosidase I (GmMan1) to the N-terminus of mRFP using the pSAT6 backbone. This segment comprises the first 49 amino acids of GmMan1 [22], including a 29-amino acid cytoplasmic tail with an 11-amino acid LCR (low complexity region) domain, a 16-amino acid transmembrane domain, and a 4-amino acid luminal coiled-coil region (Fig. S5E–G). The Man49-mRFP fusion protein localized to the *cis*-Golgi apparatus, as expected (Fig. 3E).

Therefore, we developed a transient transformation system for TKS protoplasts to verify the subcellular localizations of proteins. We evaluated several commonly used subcellular localization markers and confirmed their characteristic localization patterns in TKS protoplasts, thereby validating their utility for studying the subcellular localizations of proteins of interest.

### 2.4 Exploring transcriptional activity in TKS protoplasts

**Fig. 4:**
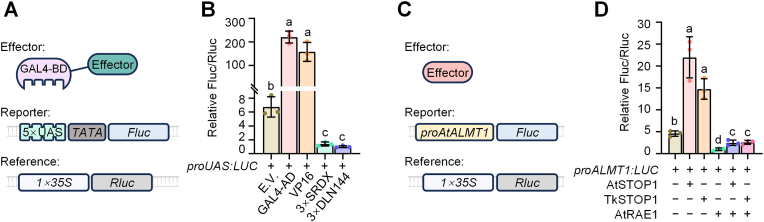
Exploring transcriptional activity in TKS protoplasts. **A** Diagram of effector, reporter, and reference constructs. Transcriptional regulatory elements were fused with GAL4-BD for transcriptional activity assays with the pUAS-luc reporter. **B** Effects of different transcriptional activation domains and transcriptional repression motifs on transcriptional activity in TKS protoplasts. **C** Diagram of the effector, reporter, and reference constructs. Plant transcription factor genes were co-expressed with the pALMT1-luc reporter for transcriptional activity assays. **D** Effects of AtSTOP1 and TkSTOP1 on activating the *ALMT1* promoter and RAE1 on repressing this transcriptional activation. Data are presented as the mean ± standard deviation (SD) of three independent biological replicates. Each data point represents one replicate, and different letters (a–d) indicate statistically significant differences (*P* < 0.05), as determined by multiple Student's *t-*test comparing each sample against the others.

Protoplasts offer a suitable cellular environment for the rapid assessment of transcriptional activation and repression by transcriptional regulators. Protoplasts can also serve as a platform to evaluate promoter binding and signaling pathways. Here, we used a *Fluc* reporter system driven by five tandem repeats of the GAL4 upstream activation sequence (UAS) and a *mini35S* promoter (Fig. S6A) to generate the pUAS-LUC vector, which we used to validate the transcriptional activities of several effectors; *Rluc* driven by the standard *35S* promoter was used as an internal reference. These effectors included transcriptional activators such as GAL4-AD and VP16 [23,24] and transcriptional repressors such as 3 × SRDX and 3 × DLN144 [25,26]. Each effector was fused to the DNA-binding domain of the yeast transcriptional activator GAL4 (GAL4-BD) and its expression controlled by the *2* × *35S* promoter (Fig. S6B–E). We performed transactivation assays by co-expressing these effectors with the reporter system in TKS protoplasts (Fig. 4A). The transcriptional activators GAL4-AD and VP16 induced 32.9-fold and 23.5-fold higher expression than the empty vector control (EV), respectively. By contrast, the transcriptional repressors 3 × SRDX and 3 × DLN144 decreased expression levels to 20.7% and 14.8% of control levels, respectively (Fig. 4B). These results indicate that TKS protoplasts provide a suitable system for studying the roles of *trans*-acting transcriptional regulators and suggest that the elements we tested could be utilized for transcriptional regulation in TKS.

We also investigated the activation of target genes by plant transcriptional activators in TKS protoplasts using a reporter system. We constructed the pALMT-LUC vector, in which the *Fluc* reporter was driven by the *AtALMT1* promoter; *Rluc* served as an internal reference. Following co-transformation with the effectors, we quantified relative luminescence to assess the binding of the effectors to the target promoters and promoter activation (Fig. 4C). Both AtSTOP1 and TkSTOP1 significantly enhanced *AtALMT1* expression in TKS protoplasts (Fig. 4D). We also explored the activity of AtRAE1, an E3 ubiquitin ligase known to interact with AtSTOP1 for its ubiquitination and degradation in *Arabidopsis* [27]. Co-transformation with *AtRAE1* resulted in a decrease in *AtALMT1* expression. Importantly, the presence of AtRAE1 prevented the activation of *AtALMT1* expression even in the presence of AtSTOP1 or TkSTOP1 (Fig. 4D). These results suggest that AtRAE1 can degrade AtSTOP1 and TkSTOP1 in TKS protoplasts, confirming the applicability of TKS protoplasts for studying plant signaling pathways.

### 2.5 Evaluating gene knockout tools using TKS protoplasts

**Fig. 5:**
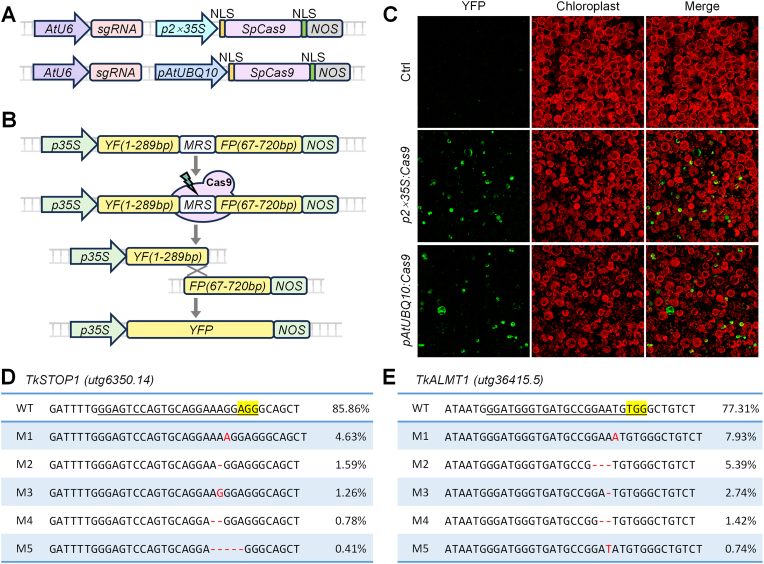
Evaluation of gene knockout tools in TKS protoplasts. **A** Construction of gene knockout vectors using the *2 × 35S* promoter and the *AtUBQ10* promoter to drive *Cas9* expression. **B** Model of the *YFFP* reporter gene and the repair mechanism after cleavage. **C** Editing of the *YFFP* reporter gene in TKS protoplasts to test the efficiency of the gene knockout tools. **D–E** Results of amplicon sequencing to test the editing of the endogenous genes *TkSTOP1* (D) and *TkALMT1* (E) in TKS protoplasts; the first five representative results are shown.

Transient expression in protoplasts provides a rapid method for evaluating the efficiency of genome editing. To develop gene knockout tools for TKS, we used the *AtU6* promoter to drive the expression of the CRISPR component sgRNA, while *SpCas9* was expressed under the control of either the *2* × *35S* or *AtUBQ10* promoter (Fig. 5A). We targeted the *YFFP* reporter gene, an engineered non-functional version of yellow fluorescent protein (*YFP*) (Fig. 5B). The *YFFP* gene consists of two *YFP* fragments with an overlapping 223 bp region interrupted by a multiple recognition site (MRS) [28]. This gene was cloned into the pSAT6 vector, allowing us to examine editing efficiency (Fig. S7). Once the MRS sequence is cleaved, creating a double-strand break, the two *YFP* fragments are capable of undergoing homology-directed repair (HDR), potentially restoring YFP activity (Fig. 5B). YFP signals were observed 18 h after co-transformation, with fluorescence recovery efficiencies of 10.9% for *2* × *35S* promoter-driven *Cas9* and 8.5% for *AtUBQ10* promoter-driven *Cas9* (Fig. 5C).

In addition, we targeted two endogenous TKS genes, *TkSTOP1* and *TkALMT1*, for knockout in the protoplast transient expression system using *2* × *35S* promoter-driven *Cas9*. We examined the frequency of mutant alleles by next-generation sequencing, which revealed editing efficiencies of 14.1% for *TkSTOP1* (Figs. 5D) and 22.7% for *TkALMT1* (Fig. 5E). These results suggest that TKS protoplasts provide an effective platform for evaluating the efficiency of gene knockout of TKS endogenous genes.

### 2.6 Evaluating base-editing tools using TKS protoplasts

In addition to gene knockout, precise base editing is also an essential technique in molecular plant breeding [29]. We previously developed a series of base-editing reporter systems using the highly sensitive NanoLUC (*Nluc*) derived from *Oplophorus gracilirostris* [30]. These systems introduce stop codons, such as TAG or TGA, that inactivate *Nlu*c and can be reactivated by base editors that correct these stop codons, leading to the recovery of Nluc luminescence. Moreover, we modified the target sequences without changing the encoded amino acids to introduce protospacer adjacent motifs (PAMs) and avoid bystander editing.

**Fig. 6:**
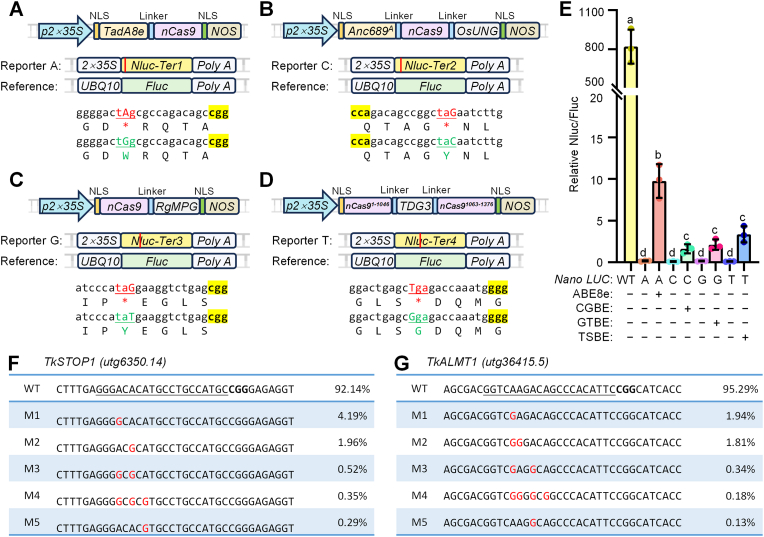
Assessment of base editors in TKS protoplasts. **A** ABE8e base editor and the corresponding reporter A. **B** CGBE base editor and the corresponding reporter C. **C** GTBE base editor and the corresponding reporter G. **D** TSBE base editor and the corresponding reporter T. **E** Expression of the base editor and the corresponding reporter in TKS protoplasts to test base-editing efficiency. Data are presented as the mean ± standard deviation (SD) of three independent biological replicates. Each data point represents one replicate, and different letters (a–d) indicate statistically significant differences (*P* < 0.05), as determined by multiple Student's *t-*test comparing each sample against the others. **F–G** Results of amplicon sequencing testing the editing of the endogenous genes *TkSTOP1* (F) and *TkALMT1* (G) in TKS protoplasts; the first five representative results are shown.

In our editing system, when the adenine base editor (ABE) [31] converts an adenine (A) to guanine (G), it changes the stop codon TAG to TGG, restoring the amino acid tryptophan (Trp) and allowing reporter A to recover Nluc luminescence (Fig. 6A, S8A). Similarly, when the C-to-G base editor (CGBE) [32] corrects the cytosine (C) in the opposite strand of the TAG codon, changing TAG to TAC and restoring the amino acid tyrosine (Tyr), luminescence from reporter C is recovered (Fig. 6B, S8B). The G-to-T base editor (GTBE) [33] also restores the amino acid Tyr by editing TAG to TAT, resulting in luminescence from reporter G (Fig. 6C, S8C). The T-to-S (G/C) base editor (TSBE) [34] changes TGA to GGA, restoring the amino acid glycine (Gly) and allowing reporter G to produce luminescence signals (Fig. 6D, S8D). When TKS protoplasts were transiently transformed with the appropriate reporter genes and the corresponding base editors, luminescence was partially recovered, indicating that base editing was functional, resulting in partially restored reporter activity (Fig. 6E).

Furthermore, we assessed the ability of ABE to perform targeted adenine base editing of the endogenous TKS genes *TkSTOP1* and *TkALMT1*. Through next-generation sequencing, we identified a variety of A-to-G base conversions within the protospacer regions, with efficiencies of 7.9% for *TkSTOP1* and 4.7% for *TkALMT1* (Fig. 6F and G). In summary, our newly developed reporter systems facilitate the testing and optimization of base editors in TKS protoplasts.

### 2.7 Genome-editing tools evaluated in TKS protoplasts show efficacy in hairy roots

**Fig. 7:**
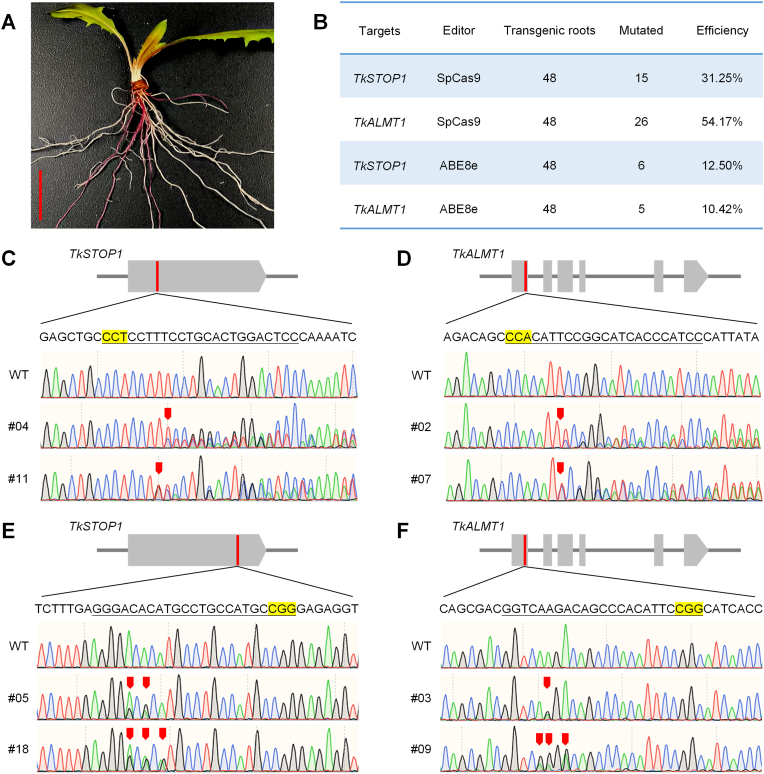
Genome-editing tools evaluated in TKS protoplasts are efficient in hairy roots. **A** Morphology of TKS hairy roots successfully induced by *Agrobacterium rhizogenes* strain K599, bar = 2 cm. **B** Summary of gene knockout and base-editing efficiencies for *TkSTOP1* and *TkALMT1* in TKS hairy roots. **C–D** Representative amplicon sequencing results of CRISPR/Cas9-mediated gene knockout of *TkSTOP1* (C) and *TkALMT1* (D) in TKS hairy roots. **E–F** Representative amplicon sequencing results of ABE8e-mediated adenine base editing of *TkSTOP1* (E) and *TkALMT1* (F) in TKS hairy roots.

Having established the utility of TKS protoplasts for rapidly evaluating genome-editing tools, we examined whether these tools would retain their efficacy in a stable, root-specific cellular context. Using the tissue-culture-free *Agrobacterium rhizogenes*-mediated hairy root transformation system previously established for TKS by our group [3], we successfully generated hairy roots using *A. rhizogenes* strain K599 (Fig. 7A). Gene knockout and base editing of *TkSTOP1* and *TkALMT1* were confirmed by amplicon sequencing, with knockout efficiencies of 31.25% and 54.17% and ABE8e-mediated A-to-G base-editing efficiencies of 12.50% and 10.42%, respectively (Fig. 7B–F); these results are consistent with the editing outcomes observed in TKS protoplasts.

## 3 Discussion

In this study, we developed an optimized, high-yielding protocol for isolating viable TKS protoplasts, laying the foundation for analyzing gene function at the cellular level in this rubber-producing species. Although an improved protocol for protoplast preparation in common dandelion (*Taraxacum officinale*) was previously developed through simple adjustments to standard enzymatic digestion conditions [15], no systematic optimization of this protocol has been conducted for rubber dandelion protoplasts, nor has their applicability for molecular biology experiments been evaluated. Considering that TKS is rich in rubber, a *cis*-polyisoprene polymer [1], leaf fragmentation might be compromised during enzymatic digestion in this plant. To maintain protoplast viability, it is critical to select young, fully expanded leaves and to remove their veins, as this procedure minimizes imbalances in osmotic pressure caused by intracellular rubber accumulation. We optimized several key factors for enzymatic digestion, including the D-mannitol concentration, enzyme composition, concentration of the antioxidant β-ME, and pH of the enzyme solution and established the optimal digestion time. Notably, the inclusion of β-ME significantly increased protoplast yield (Fig. 1K), likely due to its protective effects against damage caused by peroxides released from broken protoplasts [35]. As β-ME is both an antioxidant and an anti-apoptotic agent, it may enhance cell survival by mitigating oxidative stress. Previous studies have shown that β-ME or dithiothreitol (DTT) can improve the yield of protoplasts from fungi by rendering the cell wall more susceptible to enzymatic treatment [36]. We determined that the optimal solution for protoplast isolation from 4-week-old transplanted TKS seedlings is 1% cellulase, 0.6% macerozyme, 0.5 M D-mannitol, 20 mM KCl, 20 mM CaCl_2_, 20 mM 2-Morpholinoethanesulphonic acid (MES), and 5 mM β-ME, pH 5.6–5.8.

Selecting an efficient and stable promoter is essential for robust target gene expression in TKS protoplasts. Consistent with previous findings [17], the tandem duplication of the 250 bp upstream enhancer significantly increased gene expression in this species. Gene expression driven by the *2* × *35S* promoter was nearly 50-fold higher than that of the *mini35S* promoter and nearly 4-fold higher than that of the standard *35S* promoter (Fig. 2B). In apple (*Malus domestica*) protoplasts, the *AtUBQ10* promoter was shown to drive significantly higher gene expression than the *35S* promoter [37]. Similarly, in TKS protoplasts, the *AtUBQ10* promoter drove nearly 4-times higher gene expression compared to the standard *35S* promoter, with levels comparable to those driven by the *2 × 35S* promoter (Fig. 2B). In *Salvia miltiorrhiza* protoplasts, genome editing showed slightly higher efficiency using the tomato (*Solanum lycopersicum*) *SlEF1α* promoter than the *2 × 35S* promoter [38]. Here, gene expression in TKS protoplasts was significantly improved using the *TkEF1α* promoter compared to the standard *35S* promoter. However, the expression levels were lower than those achieved using the *AtUBQ10* and *2 × 35S* promoters (Fig. 2B). Specifically, the *TkEF1α* promoter was significantly less effective than the *AtUBQ10* and *2* × *35S* promoters (Fig. 2C and D), possibly due to species-specific differences or variations in the intrinsic strength of the *SlEF1α* and *TkEF1α* promoters. Based on these results, we selected the *2* × *35S* promoter for efficient gene expression and for examining protein localization in TKS protoplasts.

Transcriptional regulation plays a crucial role in signal transduction in plants, particularly during early responses to environmental stimuli and in stress resistance. The TKS protoplast system offers distinct advantages for transcriptional studies of regulatory dynamics within 24 h of transfection. Using the dual-luciferase reporter system, we assessed the activities of several well-characterized transcriptional activators and repressors in TKS protoplasts. Both the yeast-derived GAL4-AD transcriptional activation domain [39] and the herpes simplex virus type 1-derived VP16 activation factor [40] strongly activated transcription in TKS protoplasts. By contrast, plant-derived transcriptional repressor motifs, including SRDX and DLN144 [25,26], successfully repressed gene expression (Fig. 4B). These regulatory elements have promising applications for plant synthetic biology and for modulating gene expression, such as in non-cleaving dead Cas9 (dCas9) for CRISPR-mediated transcriptional activation (CRISPRa) or CRISPR interference (CRISPRi) systems [41,42]. Additionally, we validated the activities of plant endogenous transcription factors on target genes. In addition to confirming the binding of specific transcription factors to DNA sequences, our precisely controllable platform could be used to increase our understanding of plant signaling pathways by decoupling transcriptional regulation from systemic physiological noise.

Genome editing is a powerful tool for analyzing plant gene function and improving plant traits. In this study, we validated the efficacy of genome-editing tools, including knockout tools and base editors, in TKS protoplasts. This step is critical for optimizing such tools prior to stable transformation. This transient expression system enables the rapid assessment of editing outcomes within days. While the limited transformation efficiency and short culture time of protoplasts may result in some cells not encountering the editing tools or may not allow sufficient time for the tools to exert their effects, our results demonstrate that these tools are still highly effective. To assess the efficiency of base editing, we designed four base-editing reporter systems that restore stop codons by correcting mutated bases. Although it remains uncertain whether Nluc activity is partially restored when the stop codon is corrected to an amino acid other than the target mutation, the successful correction of stop codons confirms the functionality of our base editors in TKS protoplasts. Additionally, we confirmed the editing outcomes at the genomic level through next-generation sequencing. The system's capacity for parallel testing of multiple constructs in a single batch significantly accelerates the optimization of editing tools compared to transgenic plant workflows. Our findings suggest that transient expression in TKS protoplasts is an effective method for evaluating the efficacy of gene editing tools.

We previously established an *Agrobacterium*-mediated hairy root transformation system for TKS that does not require tissue culture, enabling stable transgene expression in a root-specific cellular environment and supporting CRISPR/Cas9-based genome editing [3]. To determine whether the editing tools optimized in the protoplast system would retain efficacy in a root-specific context, we employed this hairy root system as a complementary platform. Preliminary results confirmed successful gene knockout and base editing of *TkSTOP1* and *TkALMT1* in hairy roots (Fig. 7), resembling results in protoplasts. Together, the protoplast and hairy root systems play synergistic roles: the protoplast system enables rapid, high-throughput functional screening within 12–48 h, while the hairy root system offers stable, root-relevant validation, providing a robust framework for functional genomic research and the genetic improvement of TKS.

In summary, we established an efficient method for isolating protoplasts from TKS and developed a robust transient expression system. We successfully employed this system for promoter screening, protein expression, protein localization, and transcriptional regulation assays, laying a solid foundation for analyzing gene function and exploring signaling pathways (Fig. 7). Furthermore, we demonstrated its utility as a versatile platform for screening and optimizing genome-editing tools. Looking forward, this system could be directly employed to dissect complex metabolic pathways, such as rubber biosynthesis, to inform strategies for yield improvement. This system could also be used to test genetically engineered circuits designed to enhance stress resistance. Therefore, this work provides an indispensable cellular toolkit to accelerate the development of genome-editing tools in TKS and to bridge the gap between gene discovery and analysis of gene function in this important rubber-producing plant.

## 4 Materials and methods

### 4.1 Plant materials and growth conditions

*Taraxacum kok-saghyz* (TKS) seeds were sown in soil and cultured under a 16-h light/8-h dark cycle at 24 °C and 55–65% relative humidity. After one week, the seedlings were transplanted into individual pots. The same growth chamber conditions were maintained to prevent drought or temperature extremes. Four weeks post-transplantation, fully expanded mature leaves were harvested for protoplast isolation.

### 4.2 Protoplast isolation and transfection

An optimized TKS protoplast isolation protocol was developed based on a modified classical protocol [43]. Fully expanded leaves from 4-week-old transplanted plants were harvested, and the main veins were removed using a sharp razor blade. The leaves were cut into small pieces and digested in an enzyme solution containing 20 mM KCl (Sangon Biotech, China, A100395), 20 mM MES (Sangon Biotech, A420766; pH 5.2–6.2), a gradient of D-mannitol (PhytoTech Labs, USA, M562; 0.2–0.7 M), cellulose R-10 (Yakult Honsha, Japan, CAS: 9012-54-8; 0.5–2.0% w/v), and macerozyme R-10 (Yakult Honsha, CAS: 9032-75-1; 0.3–0.9% w/v). The enzyme solution was pre-warmed to 55 °C for 10 min, cooled to room temperature, and supplemented with 20 mM CaCl_2_ (Sigma-Aldrich, USA, C4901) and β-ME (Sangon Biotech, A355395; 0–8 mM) for digestion.

Enzymatic digestion was performed on a shaker at 40 rpm for 4–5 h at 28 °C in the dark. The digested material was filtered through a 70 μm nylon mesh (Sangon Biotech, F613462), and the filtrate was diluted with an equal volume of W5 solution composed of 2 mM MES (pH adjusted to 5.7 with KOH), 154 mM NaCl (Sangon Biotech, A100241), 125 mM CaCl_2_, and 5 mM KCl. The sample was centrifuged at 100×*g* for 5 min in a 50 mL round-bottom tube. The supernatant was discarded, and the protoplasts were gently resuspended in W5 solution. Cell morphology was examined under a fluorescence microscope, and cell counts were performed using a hematocrit counting chamber. To assess cell viability, the protoplasts were stained with 0.01% (w/v) fluorescein diacetate (FDA) for 3 min, centrifuged, washed with W5 solution, and examined by fluorescence microscopy (Ex/Em = 490/520 nm). The optimal digestion solution for protoplast isolation was determined to be 1% cellulase, 0.6% macerozyme, 0.5 M D-mannitol, 20 mM KCl, 20 mM CaCl_2_, 20 mM MES, 5 mM β-ME, with a pH range of 5.6–5.8.

For PEG/calcium-mediated transfection, protoplasts were resuspended in W5 solution and kept on ice for 15 min. After the protoplasts settled, the supernatant was carefully removed, and the protoplasts were diluted to a density of 10^6^/mL. To detect fluorescent proteins and luciferase, 100 μL of the protoplast suspension was gently mixed with 10 μL of plasmid DNA at a concentration of 1 μg/μL. An equal volume (110 μL) of PEG-CaCl_2_ solution (40% PEG4000, 0.2 M D-mannitol, 100 mM CaCl_2_) was added, and the mixture was immediately mixed gently and incubated at 28 °C for 15 min in the dark. Transfection was halted by adding 500 μL of W5 solution and centrifuging at 100×*g* for 5 min. The transfected protoplasts were washed with W5 solution, resuspended in 500 μL W5 solution, and incubated for 12–18 h as needed. When evaluating genome-editing tools, the incubation time was extended to 18–48 h. For protein expression and immunoblotting, 2 mL of protoplasts were used, and the reaction system was scaled up accordingly.

### 4.3 Plasmid construction

All plasmids used in this study were constructed in the pSAT6 backbone through a series of modifications, including promoter replacement, E3 ligase-mediated degradation system introduction, and the addition of expression cassettes (Table S1). The pDual-LUC vector was developed by separately cloning the 893 bp *2 × 35S* promoter, the 1650 bp *AtUBQ10* promoter, or the 1924 bp *TkEF1α* promoter upstream of the *LUC* coding sequence (Fig. S1). The *GFP*, *AtSTOP1* (AT1G34370), and *TkSTOP1* (evm.TU.utg6350.14) coding sequences (Fig. S2) were inserted into the pSAT6 vector using a ClonExpress II One Step Cloning Kit (Vazyme) for subsequent expression analysis. In addition, protein sorting signals or sequences from proteins with known localizations were fused to genes encoding fluorescent proteins in the pSAT6 vector to assess the subcellular localizations of the transiently expressed proteins (Figs. S4 and S5).

Using the pDual-LUC vector, the *proUAS-LUC* and *proALMT1-LUC* reporter genes were generated by replacing the *LUC* promoter. Transcriptional regulators including GAL4-AD, VP16, SRDX, DLN144, AtSTOP1, and TkSTOP1 were also cloned into the pSAT6 vector. These regulators were combined with the reporter genes to evaluate their effects on transcriptional regulation (Fig. S6). The *YFFP* reporter gene was integrated into the pSAT6 vector to visualize the results of genome editing (Fig. S7). Mutations were introduced into *Nluc*, which was then cloned into the pSAT6 vector (GenBank accession number DQ005475) (Fig. S8) and co-transformed with the gene editing tool to assess editing efficiency.

To construct the genome-editing vectors, 23-bp sequences (including the PAM) targeting *TkSTOP1* and *TkALMT1* were designed using the CRISPR-GE web server (http://skl.scau.edu.cn/targetdesign/) [44]. Synthetic oligos were annealed and inserted into the pSAT-Cas9 vector following the *AtU6* promoter via Golden Gate cloning. The base editors ABE8e, CGBE, GTBE, and TSBE (TDG3) were constructed as previously reported [33,45,46]. The target site was selected such that the corrected bases were positioned within the 3rd to 10th nucleotide from the start of the target sequence, with the PAM located at positions 21 to 23. All primers used in this study are listed in Table S1.

### 4.4 Dual-luciferase activity assay

The dual-luciferase reporter system was used for transient expression in protoplasts. Protoplasts were collected by centrifugation at 12–18 h post-transfection, and total proteins were extracted from the samples using a lysis buffer (Beyotime, RG132 M). When Fluc and Rluc were used as the reporter and internal reference, chemiluminescence was measured sequentially after the addition of luciferin (for Fluc) and coelenterazine (for Rluc), with emission wavelengths recorded at 550–580 nm and 465–495 nm, respectively. Relative expression was calculated as the ratio of Fluc to Rluc luminescence. For experiments where Nluc was the reporter and Fluc was the internal reference, chemiluminescence values were measured after the sequential addition of luciferin (for Fluc) and furimazine (for Nluc) at wavelengths of 550–580 nm and 445–475 nm, respectively. The relative expression of the reporter gene was calculated as the ratio of Nluc to Fluc luminescence.

### 4.5 Protein extraction and immunoblot analysis

Proteins were extracted from the protoplasts 12–18 h post-incubation. Protein extracts were prepared using a buffer containing 5% glycerol (Sangon Biotech, A100854), 300 mM NaCl (Sangon Biotech, A100241), 40 mM Tris-HCl (Sangon Biotech, B648002; pH 7.5), 5 mM MgCl_2_ (Sangon Biotech, A100288), 4 mM DTT (Sangon Biotech, A100281), 2 mM EDTA (Sangon Biotech, B540625; pH 8.0), and 1% (v/v) Triton X-100 (Sangon Biotech, A110694). Total proteins were extracted and denatured at 95 °C for 5 min. Immunoblotting was performed using the following primary antibodies: a mouse anti-FLAG antibody (Sangon Biotech, catalog number D191041) and a mouse anti-GFP antibody (TransGen Biotech, catalog number HT801). An HRP-conjugated secondary antibody, goat anti-mouse (Sangon Biotech, catalog number D111024), was used for detection. The reaction signal was visualized using enhanced chemiluminescence solution (Millipore, catalog number WBKLS0500).

### 4.6 Subcellular localization

For subcellular localization assays, protoplasts were harvested 12 h post-incubation. Fluorescent protein signals were detected and images captured under a ZEISS LSM980 laser-scanning confocal microscope. Using 445 nm light for excitation, CFP (cerulean) was detected and the emission light signal was collected from 470 to 500 nm; using 488 nm light for excitation, GFP was detected and the emission light signal was collected from 500 to 530 nm; using 514 nm light for excitation, YFP (Citrine) was detected and the emission light signal was collected from 525 to 555 nm; using 543 nm light for excitation, RFP was detected and the emission light signal was collected from 580 to 630 nm. To detect chloroplast autofluorescence, the emission light signal was collected from 680 to 750 nm, with no light added for excitation.

### 4.7 Detection of mutations

To evaluate genome-editing tools in the protoplast transient expression system, protoplasts were harvested 36 h post-incubation. Genomic DNA was extracted from TKS protoplasts using the CTAB method. The DNA was amplified by PCR with high-fidelity DNA polymerase and sequence-specific primers (Table S1). The resulting PCR products, which included genomic fragments containing the target loci, were subjected to high-throughput sequencing. The editing efficiency was determined by calculating the ratio of edits to total reads. The five most prevalent mutations within the protospacers, ranked by read count from the raw sequencing data, are shown in Fig. 5D and E, and Fig. 6F and G.

### 4.8 Hairy root transformation and evaluation of genome editing

TKS hairy root transformation was performed using a tissue-culture-free protocol adapted from the cut–dip–budding (CDB) delivery system [3]. Briefly, 4-week-old TKS seedlings were cut near the shoot–root junction under non-sterile conditions. The cut site was inoculated with *Agrobacterium rhizogenes* strain K599 harboring the genome-editing constructs. Inoculated explants were cultured in vermiculite at 24 °C under a 16/8-h light/dark cycle. Hairy roots were harvested 3 weeks post-inoculation, and genomic DNA was extracted from the samples using the CTAB method. Editing efficiency was determined by amplicon sequencing as described in section 4.7.

## CRediT authorship contribution statement

**Xinbo Li:** Writing – original draft, Visualization, Methodology, Investigation, Data curation, Conceptualization. **Rundong Shen:** Visualization, Validation, Formal analysis, Data curation. **Xuesong Cao:** Resources, Investigation. **Mugui Wang:** Writing – review & editing, Funding acquisition. **Jian-Kang Zhu:** Writing – review & editing, Project administration, Funding acquisition, Conceptualization. **Yifu Tian:** Writing – review & editing, Writing – original draft, Supervision, Resources, Funding acquisition, Conceptualization.

## Declaration of competing interest

The authors declare that they have no known competing financial interests or personal relationships that could have appeared to influence the work reported in this paper.

## Data Availability

All data supporting the findings of this study are available within the paper and its Supplementary Information. Additional data are available from the corresponding author upon reasonable request.
