# Supplementary material for: A dual-platform toolkit for transient gene expression and genome editing in rubber dandelion (Taraxacum kok-saghyz)
Source: aBIOTECH. 2026 May 13;7(3):100053. doi: 10.1016/j.abiote.2026.100053 (PMC13231075; doi:10.1016/j.abiote.2026.100053)
Supplement: Multimedia component 2 [file mmc2.pdf]

Supporting Information For

A dual-platform toolkit for transient gene expression and genome editing in rubber dandelion (*Taraxacum kok-saghyz*)

Xinbo Li[a, b], Rundong Shen[a, b], Xuesong Cao[c], Mugui Wang[a], Jian-Kang Zhu[a, c, *], Yifu Tian[a, b, **]

[a] Ministry of Agriculture and Rural Affairs Key Laboratory of Gene Editing Technologies (Hainan), Institute of Crop Sciences and National Nanfan Research Institute, Chinese Academy of Agricultural Sciences, Sanya, 572024, China

[b] Yazhouwan National Laboratory, Sanya, 572024, China

[c] Institute of Advanced Biotechnology, and School of Medicine, Southern University of Science and Technology, Shenzhen, 518055, China

*Corresponding author.

**Corresponding author.

E-mail address: tianyifu@caas.cn (Y. Tian), zhujk@sustech.edu.cn (J. Zhu).

This file includes:

1. Legend of Supplementary Table

2. Supplementary Figures 1-8

Table S1. Primers used for vector construction and genotyping.

Supplementary Figures

A

CCGGTCAACATGTGGAGCACGACACACTTGTCTACTCCAAAAATATCAAAGATACAGTCTCAGAAGACCAAAGGGCAATTGAGACTTT
TCAACAAAGGGTAATATCCGGAAACCTCCTCGGATTCCATTGCCCAGCTATCTGTCACTTTATTGTGAAGATAGTGGAAAAGGAAGGT
GGCTCCTACAAATGCCATCATTGCGATAAAGGAAAGGCCATCGTTGAAGATGCCTCTGCCGACAGTGGTCCCAAAGATGGACCCCCA
CCCACGAGGAGCATCGTGGAAAAAGAAGACGTTCCAACCACGTCTTCAAAGCAAGTGGATTGATGTGATAACATGGTGGAGCACGAC
ACACTTGTCTACTCCAAAAATATCAAAGATACAGTCTCAGAAGACCAAAGGGCAATTGAGACTTTTCAACAAAGGGTAATATCCGGAAA
CCTCCTCGGATTCCATTGCCCAGCTATCTGTCACTTTATTGTGAAGATAGTGGAAAAGGAAGGTGGCTCCTACAAATGCCATCATTGC
GATAAAGGAAAGGCCATCGTTGAAGATGCCTCTGCCGACAGTGGTCCCAAAGATGGACCCCCACCCACGAGGAGCATCGTGGAAAA
AGAAGACGTTCCAACCACGTCTTCAAAGCAAGTGGATTGATGTGATATCTCCACTGACGTAAGGGATGACGCACAATCCCACTATCCT
TCGCAAGACCCTTCCTCTATATAAGGAAGTTCATTTCATTTGGAGAGGACGTCGAGAGTTCTCAACACAACATATACAAAACAAACGAA
TCTCAAGCAATCAAGCATTCTACTTCTATTGCAGCAATTTAAATCATTTCTTTTAAAGCAAAAGCAATTTTCTGAAAATTTTCACCATTTA
CGAACGATAGCC

B

TTACCACAGCTCTTGCACCTAACCATAACACCTTCCCTGTATGATCGCGAAGCACCCACCCTAAGCCACATTTTAATCCTTCTGTTGGC
CATGCCCCATCAAAGTTGCACTTAACCCAAGATTGTGGTGGAGCTTCCCATGTTTCTCGTCTGTCCCGACGGTGTTGTGGTTGGTGC
TTTCCTTACATTCTGAGCCTCTTTCCTTCTAATCCACTCATCTGCATCTTCTTGTGTCCTTACTAATACCTCATTGGTTCCAAATTCCCTC
CCTTTAAGCACCAGCTCGTTTCTGTTCTTCCACAGCCTCCCAAGTATCCAAGGGACTAAAGCCTCCACATTCTTCAGATCAGGATATTC
TTGTTTAAGATGTTGAACTCTATGGAGGTTTGTATGAACTGATGATCTAGGACCGGATAAGTTCCCTTCTTCATAGCGAACTTATTCAA
AGAATGTTTTGTGTATCATTCTTGTTACATTGTTATTAATGAAAAAATATTATTGGTCATTGGACTGAACACGAGTGTTAAATATGGACC
AGGCCCCAAATAAGATCCATTGATATATGAATTAAATAACAAGAATAAATCGAGTCACCAAACCACTTGCCTTTTTAACGAGACTTGTT
CACCAACTTGATACAAAAGTCATTATCCTATGCAAATCAATAATCATACAAAAATATCCAATAACACTAAAAAATTAAAAGAAATGGATAA
TTTCACAATATGTTATACGATAAAGAAGTTACTTTTCCAAGAAATTCACTGATTTTATAAGCCCACTTGCATTAGATAAATGGCAAAAAAA
AACAAAAAGGAAAAGAAATAAAGCACGAAGAATTCTAGAAAATACGAAATACGCTTCAATGCAGTGGGACCCACGGTTCAATTATTGC
CAATTTTCAGCTCCACCGTATATTTAAAAAATAAAACGATAATGCTAAAAAAATATAAATCGTAACGATCGTTAAATCTCAACGGCTGGA
TCTTATGACGACCGTTAGAAATTGTGGTTGTCGACGAGTCAGTAATAAACGGCGTCAAAGTGGTTGCAGCCGGCACACACGAGTCGT
GTTTATCAACTCAAAGCACAAATACTTTTCCTCAACCTAAAAATAAGGCAATTAGCCAAAAACAACTTTGCGTGTAAACAACGCTCAATA
CACGTGTCATTTTATTATTAGCTATTGCTTCACCGCCTTAGCTTTCTCGTGACCTAGTCGTCCTCGTCTTTTCTTCTTCTTCTTCTATAAA
ACAATACCCAAAGAGCTCTTCTTCTTCACAATTCAGATTTCAATTTCTCAAAATCTTAAAAACTTTCTCTCAATTCTCTCTACCGTGATCA
AGGTAAATTTCTGTGTTCCTTATTCTCTCAAAATCTTCGATTTTGTTTTCGTTCGATCCCAATTTCGTATATGTTCTTTGGTTTAGATTCT
GTTAATCTTAGATCGAAGACGATTTTCTGGGTTTGATCGTTAGATATCATCTTAATTCTCGATTAGGGTTTCATAGATATCATCCGATTT
GTTCAAATAATTTGAGTTTTGTCGAATAATTACTCTTCGATTTGTGATTTCTATCTAGATCTGGTGTTAGTTTCTAGTTTGTGCGATCGA
ATTTGTCGATTAATCTGAGTTTTTCTGATTAACAG

C

GACTGACAATTTGGTACACGATACAAATAACATGACATGAACACGACACGAGTTTTCCGTGTTCGTGTTTGACAAATTCGTGTTTATTC
GTGTCGTATTCGTGTTTGGTGCGTGTTGTGTTCGTGTTTAATACCTCAGACACGAATTAAAATTCGTGTTTGCCAAATCCGTGTCGTGT
AAATTCGTGTCTGACACGAATACACAACACGCAGACACAGATTGTCAGGTCTAGTATTTATAGTTTGTTTTAGGACAAATTGTGTAACA
ATCTTTTGCGAGCCCTAAGCTTTTTAAACTTTTGACAAAACTCAAATTAAAAAAAACATGTGTGAAAATATAAGATTAGAATTTTTGTTGT
TAACATAAACTTTTAATAAAAAAACAATTTCAATTAGCATTTAACAAAAAATTAATACATGTTATATTAATTTTATTAGATTATATGTAAAAA
TTCATCTATATATCTGTCTACATTCCTCAAAAATCATTTTATATAACTTTCATATTTAATATTTTCAACTAATTTTTCCAGTCATTTTTCTCA
AAATCACATATAAAGCAAAAACGAACATTTTTCTCGACATTAACATATTCAGCAATACATTTTTAAGCTACAGTTATAAGCTTCATGTTAC
TTTGACAAAAAAATATTTTTTCTTATACAAACTGATATATCAATACTTTTAGATAATTATAAGTCAATGCCTAGAGAGCCTCTCAAGACTT
GTTATTTACCTGATTTTACCTCTATACATACATGAAGGTATTTATGGATCTTGATTCATGTGGGCAAGACTGGAACCTTTGAGAGGCCC
AAATATGCGACCAAAATATGACCGAGGGCATGAGTTGATATTAATGAAACTTGACAAGGATAATACCGTCATTAAATAACTCTTCTATA
CCTAAAAGCCAATAATAGTAAACCAAAAAAAGATGGAGGCTGGCTAGGGCATCACTATATGGAATCCACGGCTGATCCCAAACCCTCA
CTCGCACAACCGTTAGATTGCTTTATCTGACGGCTCTCAAAAAACCCTAATCTCATAGCTTCTTTACTCCACTTATAAAAACTCTCTTTG
CAGCTCACTCCTTCCATTCTTCATTTTCCTCCATCTATCGAGAGAGCTAGTCTGCAGATAATTCATTAAGGTCAGAGCAATTCTTCCGT
TTTTTCTTCTTGATTCTAGTAGATATAGTCTGTTAGCATCTATGCTTTTGTAGATCTGCTTTGATTTTAGTCTTCTAGGCCTGGTTTGTTG
TCTGATGAACCTTTATCTGTGGATGTTCTTGCTCTTGAATATATGTATATCTACATGTATGCTAGAAGTGAAACTTTTCTTGATTGATTCT
GATCGATCTTGTACAAGTTCTTTTGTTTTCGATTCTGATATCAACATAGGTTTTGTTATTTGTGAAAACCACTTTTATTTCTTGTTAAAAC
CTTTTAGATTCGACAATCGCACATGGATTTTGTAATTAATTGATGAATTCTTTCAGATATAATTGATATCTTTTTATTAAGCTGTTTTTGG
TCTATAATCACTCGATTTTGATTATTATTGTAGATCTGTGTTTTTAAAAAAGTAAACTTGTTTGATTGGTAATAATAAAAGCTAAACATCA
TGTTTTTGCTTTTCTACTTCAAGTTTTTCTAACTGATGTAGTTTTAAAACCCGATTTCGATCCTTTTACTTCACATTTATGCTGTTTTGTGA
TGTAACTTTACATATCTGGTAAAAATACTTTTATATGTTTATATGTCCTACTACTATATATATAAGTTCAATTATTTAACCATATTAAGAAC
TATTTCCTGATTAGTTACTTGCTCACATCTGTTAGTTATTTTCAAATGTGATTATTTTTACATACCTTTTCTTTATTTTAGTTTTTATCTAAC
ATTTTGTTTTAATTTATCAGG

Fig. S1 Potential highly expressed promoter sequences. **A** The *2×35S* promoter used in this study, with two tandem repeat enhancers shown in green and the *mini35S* region shown in blue. **B** The *Arabidopsis* AtUBQ10 (AT4G05320) promoter, with a 1650 bp fragment selected for use in this study. **C** The *Taraxacum kok-saghyz* TkEF1α (evm.TU.utg10321.5) promoter, with a 1924 bp fragment selected for use in this study.

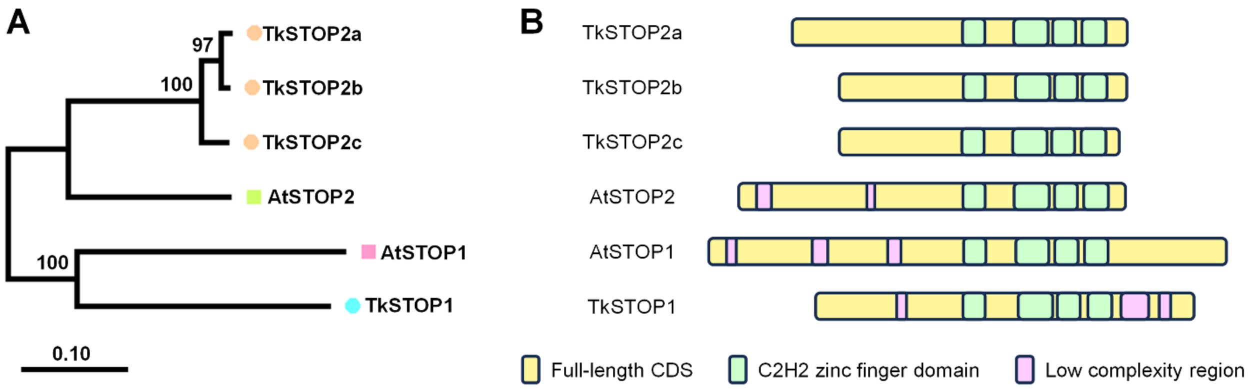

C

>TkSTOP1 (evm.TU.utg6350.14)
ATGGATCCCGACGGACTTCCTCAGCCGCTCATCAACCTCTCAAAGGTACAATCACGAATGGACCACATCCACCGCTTCCTCTCCGCTT
CACTCAACTCCAATTCCATCATTGGAGAATCGCAGATGGATATTGTCTCTAAGGAGATCGCATCCGCAATCAACCAAGTAATTGTCAA
CGGAGCTGCCCTCCTTTCCTGCACTGGACTCCCAAAATCAAAATCGGAAACCCCAAACCTTTCCTTACCGCCGACAACTTTGACTTTT
GATTCGAAACACTCGCCGGAATCTGATTTGGGTTTGAATCACTCGAAGCACGATGTTGAGTTAGGGTTTGCGAAAATCGAGGTTTTCG
GTGAGGAAGCTGGAGATGATTGGGATATCGTAGAGCTTGATGCTGTGGAGCTCCTAGCTGAACACTTACATTTCTGTGATCTTTGCG
GTAAGGGTTTCAAACGAGATGCGAATCTGCGAATGCACATGAGAGCTCACGGCAACAAATTCAAAACCCTCGAGGCGCTTGCGAAGC
CTGAGAAATCAGGATTGTCTTCTTCTTCTGAGTCGACTCGTGGTGGTAGAACGAGGTTTTCGTGCCCATTTGCTGGTTGTACCCGGAA
CAAGCTGCACAAAAAGTTCAGGCCCTTGAAATCCGTGATCTGTGTCAAAAATCACTTCAAGAGAAGCCATTGCCCCAAAATGTATTCC
TGCAATCGATGCCATAAGAAGAACTTTTCCGTACTGGCGGATCTGAAAAGCCATCTGAAGCATTGTGGTGAGACAAAATGGAAATGTT
CATGTGGGACAAGTTTTTCAAGGAAGGATAAGTTGTTTGGGCACATGGCTCTCTTTGAGGGACACATGCCTGCCATGCCGGGAGAG
GTGGTGGCGGAGGAGGATGAGAAGGCAAAAGAGGTGGCACCGGCGGCAGTGGTGGTGGAAGATGGTGGTGGTAAGGTTGGGAAG
GGGATGGAATGGATCGATAACAATATTGATGATGGTTTTTTCGATGGATTGGGTTCACTTGATGATGAGTTTTGCATGCAGGAACTTA
TTGGTTCTTCAAATGGTGGTTTCGAATGGAATTCATAG

Fig. S2 STOP1 homologs in *Taraxacum kok-saghyz*. **A** Evolutionary distances of STOP1-like proteins in *Taraxacum kok-saghyz* were analyzed using MEGA11 with the Neighbor-Joining method. Branch lengths are drawn to scale, showing the relationships among AtSTOP1 (AT1G34370), AtSTOP2 (AT5G22890), TkSTOP1 (evm.TU.utg6350.14), TkSTOP2a (evm.TU.utg6821.3), TkSTOP2b (evm.TU.utg23611.1), and TkSTOP2c (evm.TU.utg3497.3). **B** Structural domain analysis of STOP1-like proteins was performed using the SMART software. **C** Coding sequence of the *TkSTOP1* used in this study.

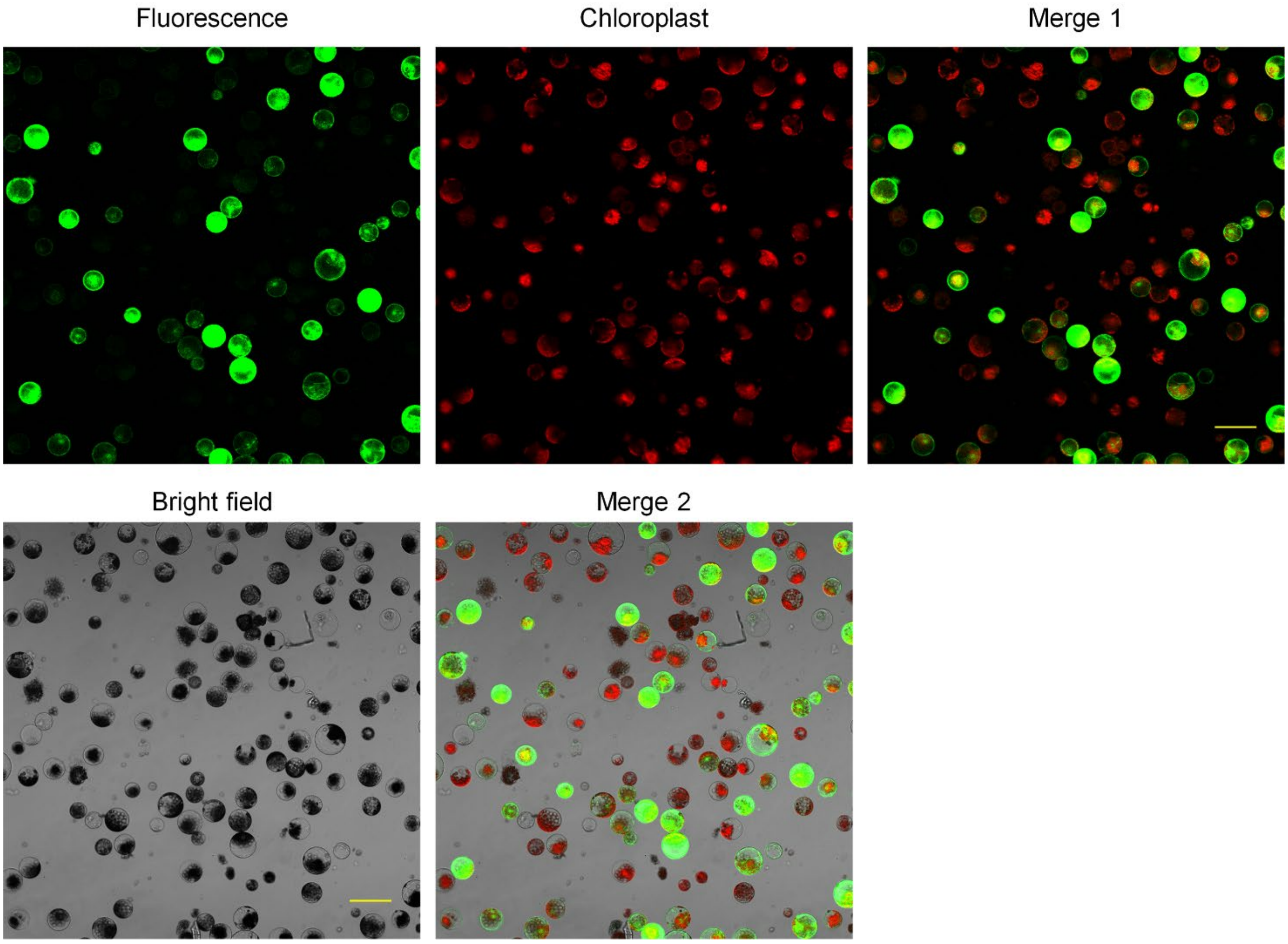

Fig. S3 Transfection efficiency in TKS protoplasts. Image shows the expression of GFP in TKS protoplasts. Transformation efficiency can be estimated by bright-field microscopy and counting of GFP-expressing cells. Bar = 100 μm.

A

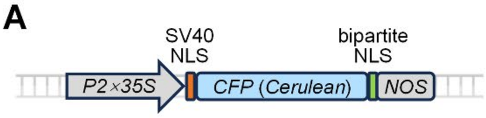

B

ATGGCCCCAAAGAAGAAGCGGAAGGTCGGTATCCACGGAGTCCCAGCAGCCATGGTGAGCAAGGGCGAGGAGCTGTTCACCGGG
GTGGTGCCCATCCTGGTCGAGCTGGACGGCGACGTAAACGGCCACAAGTTCAGCGTGTCCGGCGAGGGCGAGGGCGATGCCACC
TACGGCAAGCTGACCCTGAAGTTCATCTGCACCACCGGCAAGCTGCCCGTGCCCTGGCCCACCCTCGTGACCACCCTGACCTGGGG
CGTGCAGTGCTTCGCCCGCTACCCCGACCACATGAAGCAGCACGACTTCTTCAAGTCCGCCATGCCCGAAGGCTACGTCCAGGAGC
GCACCATCTTCTTCAAGGACGACGGCAACTACAAGACCCGCGCCGAGGTGAAGTTCGAGGGCGACACCCTGGTGAACCGCATCGA
GCTGAAGGGCATCGACTTCAAGGAGGACGGCAACATCCTGGGGCACAAGCTGGAGTACAACGCCATCAGCGACAACGTCTATATCA
CCGCCGACAAGCAGAAGAACGGCATCAAGGCCAACTTCAAGATCCGCCACAACATCGAGGACGGCAGCGTGCAGCTCGCCGACCA
CTACCAGCAGAACACCCCCATCGGCGACGGCCCCGTGCTGCTGCCCGACAACCACTACCTGAGCACCCAGTCCGCCCTGAGCAAA
GACCCCAACGAGAAGCGCGATCACATGGTCCTGCTGGAGTTCGTGACCGCCGCCGGGATCACTCTCGGCATGGACGAGCTGTACA
AGAAAAGGCCGGCGGCCACGAAAAAGGCCGGCCAGGCAAAAAAGAAAAAGTAA

C

MAPKKKRKVGIHGVPAAMVSKGEELFTGVVPILVELDGDVNGHKFSVSGEGEGDATYGKLTLKFICTTGKLPVPWPTLVTTLTWGVQCFAR
YPDHMKQHDFFKSAMPEGYVQERTIFFKDDGNYKTRAEVKFEGDTLVNRIELKGIDFKEDGNILGHKLEYNAISDNVYITADKQKNGIKANFK
IRHNIEDGSVQLADHYQQNTPIGDGPVLLPDNHYLSTQSALSKDPNEKRDHMVLLEFVTAAGITLGMDELYKKRPAATKKAGQAKKKK*

Fig. S4 Construction of nuclear localizations marker. **A** A cellular nuclear localization marker for TKS protoplasts was constructed by fusing two nuclear localization signals (NLS) to the CFP (Cerulean). **B-C** DNA sequence (B) and amino acid sequences (C) of NLS-CFP-NLS used in this work. The SV40 NLS, CFP (Cerulean), and bipartite NLS are indicated by the orange, blue, and green segments, respectively.

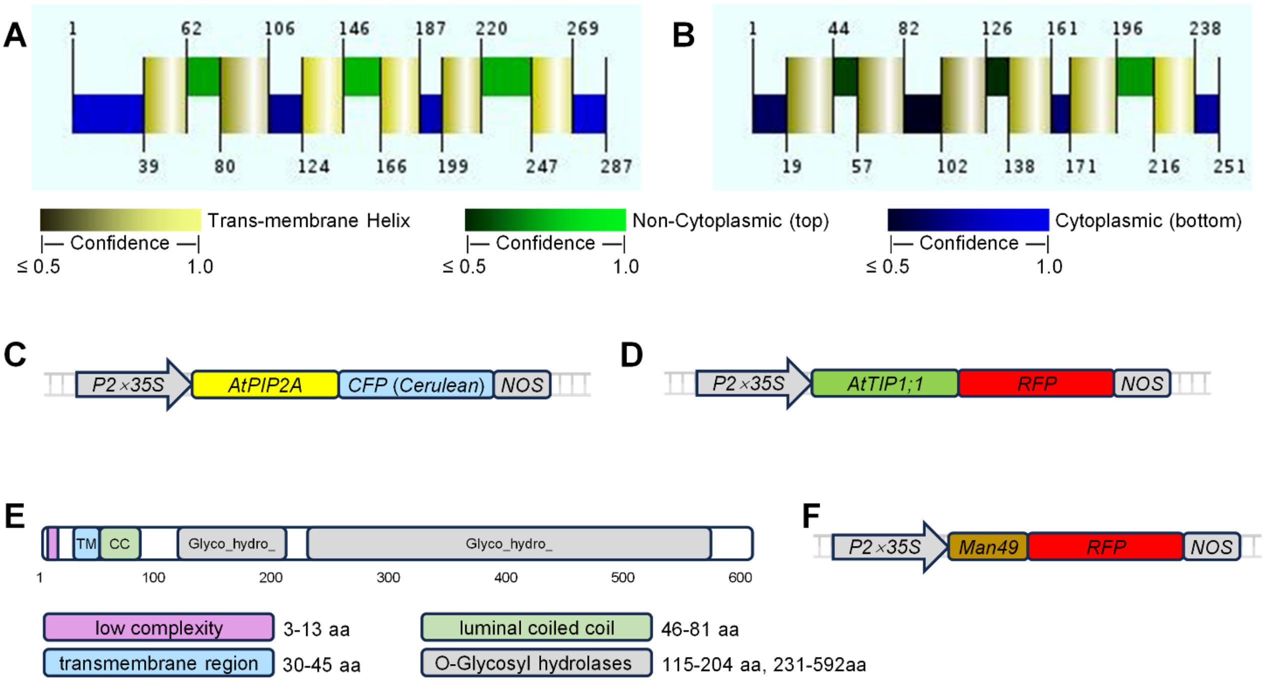

G

ATGGCGAGAGGGAGCAGATCAGTGGGTAGCAGCAGCAGCAAATGGAGGTACTGCAACCCTTCCTATTACTTGAAGCGCCCAAAGCG
TCTTGCTCTGCTCTTCATCGTTTTCGTTTGTGTCTCTTTCGTTTTCTGGGACCGTCAAACTATGGCCTCCTCCGAGGACGTCATCAAGG
AGTTCATGCGCTTCAAGGTGCGCATGGAGGGCTCCGTGAACGGCCACGAGTTCGAGATCGAGGGCGAGGGCGAGGGCCGCCCCT
ACGAGGGCACCCAGACCGCCAAGCTGAAGGTGACCAAGGGCGGCCCCCTGCCCTTCGCCTGGGACATCCTGTCCCCTCAGTTCCA
GTACGGCTCCAAGGCCTACGTGAAGCACCCCGCCGACATCCCCGACTACTTGAAGCTGTCCTTCCCCGAGGGCTTCAAGTGGGAGC
GCGTGATGAACTTCGAGGACGGCGGCGTGGTGACCGTGACCCAGGACTCCTCCCTGCAGGACGGCGAGTTCATCTACAAGGTGAA
GCTGCGCGGCACCAACTTCCCCTCCGACGGCCCCGTAATGCAGAAGAAGACCATGGGCTGGGAGGCCTCCACCGAGCGGATGTAC
CCCGAGGACGGCGCCCTGAAGGGCGAGATCAAGATGAGGCTGAAGCTGAAGGACGGCGGCCACTACGACGCCGAGGTCAAGACC
ACCTACATGGCCAAGAAGCCCGTGCAGCTGCCCGGCGCCTACAAGACCGACATCAAGCTGGACATCACCTCCCACAACGAGGACTA
CACCATCGTGGAACAGTACGAGCGCGCCGAGGGCCGCCACTCCACCGGCGCCTAA

H

MARGSRSVGSSSSKWRYCNPSYYLKRPKRLALLFIVFVCVSFVFWDRQTMASSEDVIKEFMRFKVRMEGSVNGHEFEIEGEGEGRPYEGT
QTAKLKVTKGGPLPFAWDILSPQFQYGSKAYVKHPADIPDYLKLSFPEGFKWERVMNFEDGGVVTVTQDSSLQDGEFIYKVKLRGTNFPSD
GPVMQKKTMGWEASTERMYPEDGALKGEIKMRLKLKDGGHYDAEVKTTYMAKKPVQLPGAYKTDIKLDITSHNEDYTIVEQYERAEGRHS
TGA*

Fig. S5 Construction of plasma membrane, tonoplast and cis-Golgi localization markers. **A-B** Transmembrane domain prediction of (A) AtPIP2A (AT3G53420), and (B) AtTIP1;1 (AT2G36830). **C-D** Schematic of the fusion constructs used to generate the plasma membrane marker AtPIP2A-CFP (C) and the tonoplast marker AtTIP1;1-RFP (D). **E** Domain architecture prediction of GmMan1 by SMART analysis, highlighting the N-terminal 49-amino-acid region (Man49) used for marker construction. **F** Design of the cis-Golgi marker (Man49-RFP). **G-H** DNA sequence (G) and amino acid sequences (H) of Man49-RFP used in this work. The red segment represents RFP, and the upstream sequence corresponds to Man49. Within Man49, the pink, blue, and green regions denote the low-complexity region, transmembrane region, and luminal coiled-coil region, respectively.

A

CGGAGTACTGTCCTCCGAGCGGAGTACTGTCCTCCGAGCGGAGTACTGTCCTCCGAGCGGAGTACTGTCCTCCGAGCGGAGTACTGTC
CTCCGAGGCTAGCCAAGACCCTTCCTCTATATAAGGAAGTTCATTTCATTTGGAGAGGATCCAGGCCTGCCATGGAAGACGCCAAAAACA
TAAAGAAAGGCCCGGCGCCATTCTATCCTCTAGAGGATGGAACCGCTGGAGAGCAACTGCATAAGGCTATGAAGAGATACGCCCTGGTT
CCTGGAACAATTGCTTTTACAGATGCACATATCGAGGTGAACATCACGTACGCGGAATACTTCGAAATGTCCGTTCGGTTGGCAGAAGCTA
TGAAACGATATGGGCTGAATACAAATCACAGAATCGTCGTATGCAGTGAAAACTCTCTTCAATTCTTTATGCCGGTGTTGGGCGCGTTATT
TATCGGAGTTGCAGTTGCGCCCGCGAACGACATTTATAATGAACGTGAATTGCTCAACAGTATGAACATTTCGCAGCCTACCGTAGTGTTT
GTTTCCAAAAAGGGGTTGCAAAAAATTTTGAACGTGCAAAAAAAATTACCAATAATCCAGAAAATTATTATCATGGATTCTAAAACGGATTAC
CAGGGATTTCAGTCGATGTACACGTTCGTCACATCTCATCTACCTCCCGGTTTTAATGAATACGATTTTGTACCAGAGTCCTTTGATCGTGA
CAAAACAATTGCACTGATAATGAATTCCTCTGGATCTACTGGGTTACCTAAGGGTGTGGCCCTTCCGCATAGAACTGCCTGCGTCAGATTC
TCGCATGCCAGAGATCCTATTTTTGGCAATCAAATCATTCCGGATACTGCGATTTTAAGTGTTGTTCCATTCCATCACGGTTTTGGAATGTT
TACTACACTCGGATATTTGATATGTGGATTTCGAGTCGTCTTAATGTATAGATTTGAAGAAGAGCTGTTTTTACGATCCCTTCAGGATTACA
AAATTCAAAGTGCGTTGCTAGTACCAACCCTATTTTCATTCTTCGCCAAAAGCACTCTGATTGACAAATACGATTTATCTAATTTACACGAAA
TTGCTTCTGGGGGCGCACCTCTTTCGAAAGAAGTCGGGGAAGCGGTTGCAAAACGCTTCCATCTTCCAGGGATACGACAAGGATATGGG
CTCACTGAGACTACATCAGCTATTCTGATTACACCCGAGGGGGATGATAAACCGGGCGCGGTCGGTAAAGTTGTTCCATTTTTTGAAGCG
AAGGTTGTGGATCTGGATACCGGGAAAACGCTGGGCGTTAATCAGAGAGGCGAATTATGTGTCAGAGGACCTATGATTATGTCCGGTTAT
GTAAACAATCCGGAAGCGACCAACGCCTTGATTGACAAGGATGGATGGCTACATTCTGGAGACATAGCTTACTGGGACGAAGACGAACAC
TTCTTCATAGTTGACCGCTTGAAGTCTTTAATTAAATACAAAGGATATCAGGTGGCCCCCGCTGAATTGGAATCGATATTGTTACAACACCC
CAACATCTTCGACGCGGGCGTGGCAGGTCTTCCCGACGATGACGCCGGTGAACTTCCCGCCGCCGTTGTTGTTTTGGAGCACGGAAAGA
CGATGACGGAAAAAGAGATCGTGGATTACGTCGCCAGTCAAGTAACAACCGCGAAAAAGTTGCGCGGAGGAGTTGTGTTTGTGGACGAA
GTACCGAAAGGTCTTACCGGAAAACTCGACGCAAGAAAAATCAGAGAGATCCTCATAAAGGCCAAGAAGGGCGGAAAGTCCAAATTGTAA

B

ATGAAGCTACTGTCTTCTATCGAACAAGCATGCGATATTTGCCGACTTAAAAAGCTCAAGTGCTCCAAAGAAAAACCGAAGTGCGCCAAGT
GTCTGAAGAACAACTGGGAGTGTCGCTACTCTCCCAAAACCAAAAGGTCTCCGCTGACTAGGGCACATCTGACAGAAGTGGAATCAAGGC
TAGAAAGACTGGAACAGCTATTTCTACTGATTTTTCCTCGAGAAGACCTTGACATGATTTTGAAAATGGATTCTTTACAGGATATAAAAGCAT
TGTTAACAGGATTATTTGTACAAGATAATGTGAATAAAGATGCCGTCACAGATAGATTGGCTTCAGTGGAGACTGATATGCCTCTAACATTG
AGACAGCATAGAATAAGTGCGACATCATCATCGGAAGAGAGTAGTAACAAAGGTCAAAGACAGTTGACTGTATCGGGAGGGGGTTCAGG
ATCCGCCAATTTTAATCAAAGTGGGAATATTGCTGATAGCTCATTGTCCTTCACTTTCACTAACAGTAGCAACGGTCCGAACCTCATAACAA
CTCAAACAAATTCTCAAGCGCTTTCACAACCAATTGCCTCCTCTAACGTTCATGATAACTTCATGAATAATGAAATCACGGCTAGTAAAATTG
ATGATGGTAATAATTCAAAACCACTGTCACCTGGTTGGACGGACCAAACTGCGTATAACGCGTTTGGAATCACTACAGGGATGTTTAATAC
CACTACAATGGATGATGTATATAACTATCTATTCGATGATGAAGATACCCCACCAAACCCAAAAAAAGAGTAG

C

ATGAAGCTACTGTCTTCTATCGAACAAGCATGCGATATTTGCCGACTTAAAAAGCTCAAGTGCTCCAAAGAAAAACCGAAGTGCGCCAAGT
GTCTGAAGAACAACTGGGAGTGTCGCTACTCTCCCAAAACCAAAAGGTCTCCGCTGACTAGGGCACATCTGACAGAAGTGGAATCAAGGC
TAGAAAGACTGGAACAGCTATTTCTACTGATTTTTCCTCGAGAAGACCTTGACATGATTTTGAAAATGGATTCTTTACAGGATATAAAAGCAT
TGTTAACAGGATTATTTGTACAAGATAATGTGAATAAAGATGCCGTCACAGATAGATTGGCTTCAGTGGAGACTGATATGCCTCTAACATTG
AGACAGCATAGAATAAGTGCGACATCATCATCGGAAGAGAGTAGTAACAAAGGTCAAAGACAGTTGACTGTATCGGGAGGGGGTTCAGG
ATCCGCCCCCCCGACCGATGTCAGCCTGGGGGACGAGCTCCACTTAGACGGCGAGGACGTGGCGATGGCGCATGCCGACGCGCTAGA
CGATTTCGATCTGGACATGTTGGGGGACGGGGATTCCCCGGGTCCGGGATTTACCCCCCACGACTCCGCCCCCTACGGCGCTCTGGAT
ATGGCCGACTTCGAGTTTGAGCAGATGTTTACCGATGCCCTTGGAATTGACGAGTACGGTGGGTAG

D

ATGAAGCTACTGTCTTCTATCGAACAAGCATGCGATATTTGCCGACTTAAAAAGCTCAAGTGCTCCAAAGAAAAACCGAAGTGCGCCAAGT
GTCTGAAGAACAACTGGGAGTGTCGCTACTCTCCCAAAACCAAAAGGTCTCCGCTGACTAGGGCACATCTGACAGAAGTGGAATCAAGGC
TAGAAAGACTGGAACAGCTATTTCTACTGATTTTTCCTCGAGAAGACCTTGACATGATTTTGAAAATGGATTCTTTACAGGATATAAAAGCAT
TGTTAACAGGATTATTTGTACAAGATAATGTGAATAAAGATGCCGTCACAGATAGATTGGCTTCAGTGGAGACTGATATGCCTCTAACATTG
AGACAGCATAGAATAAGTGCGACATCATCATCGGAAGAGAGTAGTAACAAAGGTCAAAGACAGTTGACTGTATCGGGAGGGGGTTCAGG
ATCCCTTGATCTTGATCTTGAACTTAGACTTGGTTTTGCTGGAAGTCTAGACCTGGACCTCGAGCTCCGGCTCGGATTCGCGGGGTCGCT
TGACCTAGACCTGGAACTACGACTAGGGTTTGCATAG

E

ATGAAGCTACTGTCTTCTATCGAACAAGCATGCGATATTTGCCGACTTAAAAAGCTCAAGTGCTCCAAAGAAAAACCGAAGTGCGCCAAGT
GTCTGAAGAACAACTGGGAGTGTCGCTACTCTCCCAAAACCAAAAGGTCTCCGCTGACTAGGGCACATCTGACAGAAGTGGAATCAAGGC
TAGAAAGACTGGAACAGCTATTTCTACTGATTTTTCCTCGAGAAGACCTTGACATGATTTTGAAAATGGATTCTTTACAGGATATAAAAGCAT
TGTTAACAGGATTATTTGTACAAGATAATGTGAATAAAGATGCCGTCACAGATAGATTGGCTTCAGTGGAGACTGATATGCCTCTAACATTG
AGACAGCATAGAATAAGTGCGACATCATCATCGGAAGAGAGTAGTAACAAAGGTCAAAGACAGTTGACTGTATCGGGAGGGGGTTCAGG
ATCCGATTTGAACAGTCCACCAGGAAGTGACCTCAATTCACCTCCGGGCTCAGATCTGAATAGTCCACCCTAG

Fig. S6 Construction of the pUAS-LUC and different transcriptional effectors. **A** Sequence of the pUAS-LUC reporter, with the *5×UAS* sequence shown in green, the *TATA-box* region derived from *mini35S* shown in red and the *Fluc* sequence shown in yellow. **B** GAL4-BD (in green) is expressed in fusion with the transcriptional activator GAL4-AD (in red) via a linker. **C** GAL4-BD (in green) is expressed in fusion with the transcriptional activator VP16 (in red) via a linker. **D** GAL4-BD (in green) is expressed in fusion with the transcriptional repression structural domain 3×SRDX (in blue) via a linker. **E** GAL4-BD (in green) is expressed in fusion with the transcriptional repression domain 3×DLN144 (in blue) via a linker.

A

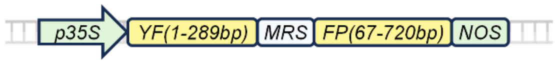

B

ATGGTGAGCAAGGGCGAGGAGCTGTTCACCGGGGTGGTGCCCATCCTGGTCGAGCTGGACGGCGACGTAAACGGCCACAAGTTCAGCGTGTCCGGCGAGGGCGAGG
GCGATGCCACCTACGGCAAGCTGACCCTGAAGTTCATCTGCACCACCGGCAAGCTGCCCGTGCCCTGGCCCACCCTCGTGACCACCTTCGGCTACGGCCTGATGTGCTT
CGCCCGCTACCCCGACCACATGAAGCAGCACGACTTCTTCAAGTCCGCCATGCCCGAAGGCTACGTCCAGGAGCACTAGTTCCCTTTATCTCTTAGGGATAACAGGGTA
ATAGAGATAAA**GGG**AGGCCTGTAAACGGCCACAAGTTCAGCGTGTCCGGCGAGGGCGAGGGCGATGCCACCTACGGCAAGCTGACCCTGAAGTTCATCTGCACCACC
GGCAAGCTGCCCGTGCCCTGGCCCACCCTCGTGACCACCTTCGGCTACGGCCTGATGTGCTTCGCCCGCTACCCCGACCACATGAAGCAGCACGACTTCTTCAAGTCCG
CCATGCCCGAAGGCTACGTCCAGGAGCGCACCATCTTCTTCAAGGACGACGGCAACTACAAGACCCGCGCCGAGGTGAAGTTCGAGGGCGACACCCTGGTGAACCGC
ATCGAGCTGAAGGGCATCGACTTCAAGGAGGACGGCAACATCCTGGGGCACAAGCTGGAGTACAACTACAACAGCCACAACGTCTATATCATGGCCGACAAGCAGAA
GAACGGCATCAAGGTGAACTTCAAGATCCGCCACAACATCGAGGACGGCAGCGTGCAGCTCGCCGACCACTACCAGCAGAACACCCCCATCGGCGACGGCCCCGTGC
TGCTGCCCGACAACCACTACCTGAGCTACCAGTCCGCCCTGAGCAAAGACCCCAACGAGAAGCGCGATCACATGGTCCTGCTGGAGTTCGTGACCGCCGCCGGGATCA
CTCTCGGCATGGACGAGCTGTACAAGTGA

Fig. S7 Construction of the *YFFP* reporter. **A** Schematic of the *YFFP* reporter gene. **B** Sequence of the *YFFP* reporter gene used in this work, with the *MRS* region highlighted in blue.

A

ATGGTCTTCACACTCGAAGATTTCGTTGGGGACTAGCGCCAGACAGC**CGG**CTACAACCTGGACCAAGTCCTTGAACAGGGAGGTGT
GTCCAGTTTGTTTCAGAATCTCGGGGTGTCCGTAACTCCGATCCAAAGGATTGTCCTGAGCGGTGAAAATGGGCTGAAGATCGACAT
CCATGTCATCATCCCGTATGAAGGTCTGAGCGGCGACCAAATGGGCCAGATCGAAAAAATTTTTAAGGTGGTGTACCCTGTGGATGA
TCATCACTTTAAGGTGATCCTGCACTATGGCACACTGGTAATCGACGGGGTTACGCCGAACATGATCGACTATTTCGGACGGCCGTA
TGAAGGCATCGCCGTGTTCGACGGCAAAAAGATCACTGTAACAGGGACCCTGTGGAACGGCAACAAAATTATCGACGAGCGCCTGA
TCAACCCCGACGGCTCCCTGCTGTTCCGAGTAACCATCAACGGAGTGACCGGCTGGCGGCTGTGCGAACGCATTCTGGCGTAA

B

ATGGTCTTCACACTCGAAGATTTCGTTGGGGACTGGCG**CCA**GACAGCCGGCTAGAATCTTGACCAAGTCCTTGAACAGGGAGGTGT
GTCCAGTTTGTTTCAGAATCTCGGGGTGTCCGTAACTCCGATCCAAAGGATTGTCCTGAGCGGTGAAAATGGGCTGAAGATCGACAT
CCATGTCATCATCCCGTATGAAGGTCTGAGCGGCGACCAAATGGGCCAGATCGAAAAAATTTTTAAGGTGGTGTACCCTGTGGATGA
TCATCACTTTAAGGTGATCCTGCACTATGGCACACTGGTAATCGACGGGGTTACGCCGAACATGATCGACTATTTCGGACGGCCGTA
TGAAGGCATCGCCGTGTTCGACGGCAAAAAGATCACTGTAACAGGGACCCTGTGGAACGGCAACAAAATTATCGACGAGCGCCTGA
TCAACCCCGACGGCTCCCTGCTGTTCCGAGTAACCATCAACGGAGTGACCGGCTGGCGGCTGTGCGAACGCATTCTGGCGTAA

C

ATGGTCTTCACACTCGAAGATTTCGTTGGGGACTGGCGACAGACAGCCGGCTACAACCTGGACCAAGTCCTTGAACAGGGAGGTGT
GTCCAGTTTGTTTCAGAATCTCGGGGTGTCCGTAACTCCGATCCAAAGGATTGTCCTGAGCGGTGAAAATGGGCTGAAGATCGACAT
CCATGTCATCATCCCATAGGAAGGTCTGAG**CGG**CGACCAAATGGGCCAGATCGAAAAAATTTTTAAGGTGGTGTACCCTGTGGATGA
TCATCACTTTAAGGTGATCCTGCACTATGGCACACTGGTAATCGACGGGGTTACGCCGAACATGATCGACTATTTCGGACGGCCGTA
TGAAGGCATCGCCGTGTTCGACGGCAAAAAGATCACTGTAACAGGGACCCTGTGGAACGGCAACAAAATTATCGACGAGCGCCTGA
TCAACCCCGACGGCTCCCTGCTGTTCCGAGTAACCATCAACGGAGTGACCGGCTGGCGGCTGTGCGAACGCATTCTGGCGTAA

D

ATGGTCTTCACACTCGAAGATTTCGTTGGGGACTGGCGACAGACAGCCGGCTACAACCTGGACCAAGTCCTTGAACAGGGAGGTGT
GTCCAGTTTGTTTCAGAATCTCGGGGTGTCCGTAACTCCGATCCAAAGGATTGTCCTGAGCGGTGAAAATGGGCTGAAGATCGACAT
CCATGTCATCATCCCGTATGAAGGACTGAGCTGAGACCAAATG**GGG**CAGATCGAAAAAATTTTTAAGGTGGTGTACCCTGTGGATGA
TCATCACTTTAAGGTGATCCTGCACTATGGCACACTGGTAATCGACGGGGTTACGCCGAACATGATCGACTATTTCGGACGGCCGTA
TGAAGGCATCGCCGTGTTCGACGGCAAAAAGATCACTGTAACAGGGACCCTGTGGAACGGCAACAAAATTATCGACGAGCGCCTGA
TCAACCCCGACGGCTCCCTGCTGTTCCGAGTAACCATCAACGGAGTGACCGGCTGGCGGCTGTGCGAACGCATTCTGGCGTAA

Fig. S8 Sequence of mutated Nluc coding genes in base editors. **A** Sequence of reporter A generated by mutating *Nluc* for testing ABE8e, with the sgRNA target A highlighted in blue and the PAM sequence bolded. **B** Generation of reporter C sequence for testing CGBE by mutating *Nluc*, with the sgRNA target C highlighted in blue and the PAM sequence bolded. **C** Generation of reporter G sequence for testing GTBE by mutation of *Nluc*, with sgRNA target G highlighted in blue and the PAM sequence bolded. **D** Generation of reporter T sequence for testing TSBE by mutation of *Nluc*, with the sgRNA target T highlighted in blue and the PAM sequence bolded.
